# Letter to the editor: Römer et al. The significance of transcervical ultrasound-guided radiofrequency ablation in the treatment of symptomatic fibroids: results of an expert consensus from German speaking countries? In the Archives of Gynecology and Obstetrics volume 306, pages 1–6 (2022)

**DOI:** 10.1007/s00404-022-06761-4

**Published:** 2022-11-17

**Authors:** Matthias David

**Affiliations:** Klinik für Gynäkologie, Campus Virchow-Klinikum, Charité – Universitätsmedizin Berlin, Augustenburger Platz 1 D, 13353 Berlin, Germany

Roemer et al. have published the important tconsensus paper “The significance of transcervical ultrasound-guided radiofrequency ablation in the treatment of symptomatic fibroids: results of an expert consensus from German-speaking countries” in the Archives of Gynecology and Obstetrics volume 306, pages 1–6 (2022).

This procedure is not quite new, but an interesting addition to the range of therapies, especially for FIGO type 2 and 3 fibroids resp. by these related complaints.

I do not want to comment on the content, but as far as I know, it has not been customary for the medical director of the company that manufactures the medical device under discussion to be listed as the last author.

This is a significant conflict of interest which, in my view, has a decisive influence on and devalues the paper. We have a duty, also to the fibroid patients seeking advice, to state this precisely.

In the interests of transparency and scientific fairness, it should be explained whether the company Gynesonics, Redwood City, CA, USA also financed the consensus meeting and/or took over the wording of the article or the revision of the English version, for example.

